# Third-line treatment patterns in HER2-positive metastatic breast cancer: a retrospective analysis of real-world data in Canada

**DOI:** 10.3389/jpps.2023.12078

**Published:** 2023-12-13

**Authors:** Karen Gambaro, Mélanie Groleau, Suzan McNamara, Arif Awan, Maged Salem, Mahmoud Abdelsalam, Eve St-Hilaire, François Vincent, Julie Carrier, Helen MacKay, Louise Provencher, Dominique Boudreau, Zineb Hamilou, Fred Saad, Cristiano Ferrario, Gerald Batist, Maud Marques

**Affiliations:** ^1^ Canadian National Centres of Excellence-Exactis Innovation, Montreal, QC, Canada; ^2^ Knight Therapeutics Inc., Montreal, QC, Canada; ^3^ The Ottawa Hospital, Ottawa, ON, Canada; ^4^ Horizon Health Network-The Moncton Hospital, Moncton, NB, Canada; ^5^ Centre Hospitalier Universitaire Dr. Georges-L.-Dumont, Moncton, NB, Canada; ^6^ Centre Hospitalier Régional de Trois-Riviéres, Trois-Riviéres, QC, Canada; ^7^ Centre Hospitalier Universitaire de Sherbrooke, Sherbrooke, QC, Canada; ^8^ Odette Cancer Centre, Sunnybrook Health Sciences Centre, Toronto, ON, Canada; ^9^ Centre Hospitalier Universitaire de Québec, Québec City, QC, Canada; ^10^ Centre Hospitalier Universitaire de Montréal, Montreal, QC, Canada; ^11^ Segal Cancer Centre-Jewish General Hospital, Montreal, QC, Canada

**Keywords:** real-world data, real-world evidence, HER2+ metastatic breast cancer, PMT registry, treatment landscape, Canada, the exactis network

## Abstract

There is an increasing demand for real-world data pertaining to the usage of cancer treatments, especially in settings where no standard treatment is specifically recommended. This study presents the first real-world analysis of third-line treatment patterns in HER2-positive metastatic breast cancer (mBC) patients in Canada. The purpose was to assess evolution of clinical practice and identify unmet needs in post-second-line therapy. Retrospective data from medical records of 66 patients who received third-line treatment before 31st October 2018, and data from 56 patients who received third-line treatment after this date, extracted from the Personalize My Treatment (PMT) cancer patient registry, were analyzed. In the first cohort, the study revealed heterogeneity in the third-line setting, with trastuzumab, lapatinib, and T-DM1 being the main treatment options. Even though data were collected before the wide availability of tucatinib, neratinib and trastuzumab deruxtecan in Canada, the PMT cohort revealed the emergence of new therapeutic combinations and a shift from lapatinib usage to T-DM1 choice was observed. These findings underscore the evolving nature of third-line treatment strategies in Canada, a facet that is intrinsically tied to the availability of new drugs. The absence of a consensus on post-second-line treatment highlights the pressing need for more efficient therapeutic alternatives beyond the currently available options. This study not only offers valuable insights into the present landscape of third-line treatment in Canada but validates the significance and effectiveness of the PMT registry as a tool for generating pan-Canadian real-world evidence in oncology and its capacity to provide information on evolution of therapeutic practices.

## Introduction

The overexpression of human epidermal growth factor receptor 2 (HER2), a transmembrane glycoprotein epidermal growth factor receptor (EGFR) with tyrosine kinase activity, is observed in 15%–20% of newly diagnosed breast cancer (BC) patients and is historically associated with a more aggressive phenotype, increased risk of recurrence, and poorer outcome [1, 2]. Over the last few decades, the introduction of new anti-HER2 therapies has dramatically improved patients’ survival, including patients with advanced and metastatic disease [3].

Diverse HER2 targeted strategies have emerged including monoclonal antibodies that bind with a high affinity to HER2 protein at the surface of the cells (trastuzumab [4], pertuzumab [5]), antibody-drug conjugates linked to a chemotherapy drug (ado-trastuzumab emtansine or T-DM1 [6] and fam-trastuzumab deruxtecan or T-DXd [7]), as well as small-molecule tyrosine kinase inhibitors (lapatinib [8], neratinib [9] and tucatinib [10]). These targeted therapies can be administered with or without chemotherapy and hormone therapy treatment, as treatment combinations have been observed to be even more effective. Dual blockade of the HER2 signaling pathway using trastuzumab and pertuzumab, in combination with chemotherapy, has shown significant benefits in the metastatic setting for patients who have not received prior anti-HER2 specific agents [11] and for patients with disease relapse after completing adjuvant treatment, as highlighted by the CLEOPATRA trial results [12].

Consequently, trastuzumab + pertuzumab and a taxane is recommended as the first-line treatment of metastatic or unresectable HER2+ BC by the National Comprehensive Cancer Network [13] (NCCN) and the European Society for Medical Oncology [14] (ESMO) guidelines. Up until 2021, the use of T-DM1 was recommended as second-line treatment as improvement in both overall survival (OS) and progression-free survival (PFS) in patients with advanced disease was observed when compared to physician’s choice treatment or lapatinib in the TH3RESA [15] and EMILIA [16] studies respectively. In 2013, Health Canada approved T-DM1 for use in the metastatic setting in patients who have received prior trastuzumab and a taxane treatment [17]. Recent critical evidence generated by the phase III DESTINY-Breast-03 trial revealed that among HER2-positive metastatic breast cancer (mBC) patients previously treated with trastuzumab and a taxane, the risk of disease progression or death was lower for patients treated with T-DXd than for those who received T-DM1 [18], resulting in a shift from T-DM1 to T-DXd as the standard second-line therapy in the metastatic setting, as recommended by the NCCN and ESMO guidelines, and a valid option for patients called “rapid progressor” presenting disease relapse within 6 months after completing adjuvant therapy.

Despite these outstanding advances, almost inevitably, patients with advanced disease, who initially respond to targeted strategies develop resistance with concomitant disease progression. As many as half of the patients with advanced disease will develop brain metastasis (BM), where standard treatment approaches are local therapies. Interestingly, the HER2CLIMB trial [19, 20] provided evidence that the overall survival (OS) of patients with BM was improved by the addition of tucatinib to trastuzumab/capecitabine, offering another option as second-line treatment for these patients. Following treatment with trastuzumab, pertuzumab and T-DM1 or T-DXd, third-line options include diverse combinations of chemo- and targeted therapy (including neratinib, tucatinib, trastuzumab, lapatinib, T-DM1), targeted therapy in dual-blockade without chemotherapy, or hormone therapy and targeted therapy for patients with hormone receptor-positive status. In 2021, the HER2CLIMB trial showed an improved OS with the combination of tucatinib + trastuzumab + capecitabine in patients with brain metastases, who had received prior trastuzumab, pertuzumab and T-DM1 and established this combination as the preferred treatment choice in this population. However, in Canada, the access to new drug being complex and dependent of Health Canada approval and provincial funding, decision making is not always aligned with international guidelines and is rather based on several factors such as available and funded drugs, international guidelines, clinical trial data and toxicity profile [21]. Access to medications through public funding can significantly vary across Canadian provinces, contributing to disparities in treatment timelines and coverage. In certain cases, differences in negotiation timelines for final pricing negotiations among provinces may result in varying effective funding dates, creating gaps of 2–6 months for drugs such as pertuzumab, T-DM1, and tucatinib. In more extreme cases, certain drugs may only receive public funding in specific provinces, further exacerbating access inequalities. For instance, while Lapatinib is reimbursed in Quebec and New Brunswick, it remains uncovered in Ontario. Nonetheless, patients have alternative avenues to access non-publicly covered medications, including private insurance, out-of-pocket expenses, or patient access programs. Within this context, there is a genuine need for real-world data (RWD) regarding the use of cancer treatments to evaluate adherence with current recommendations and the evolution of patient management in real life setting.

In 2020, Exactis Innovation launched an observational, multi-site retrospective study to capture Canadian RWD of 66 HER2+ mBC patients who started third-line therapy before 31st October 2018, through patient’s medical chart review (NCT04566458). With the objective of capturing potential evolution in third-line treatment, we extended the timeframe of our investigation and interrogated the pan-Canadian cancer patient registry “Personalize My Treatment” (PMT) (NCT02355171) in 2023 and retrospectively extracted RWD of 56 additional patients who started third-line therapy after 31st October 2018, comparing the results in the present study.

This present study is the first attempt to analyze RWD on the usage of cancer treatments in this specific setting, with the aim to uncover standard of care practices in Canada, evaluate adherence to clinical recommendations and track evolution of patient management. With these efforts, we aim to pinpoint unmet medical needs and advocate for improved access to additional cancer agents within the Canadian healthcare landscape.

## Methods

### Study design

In 2020, a chart review method was used to retrospectively collect data from patients’ medical files treated at 5 different Canadian sites (NCT04566458). This chart review study was approved by the review ethical board (REB) of each participating site, all of which are part of the Exactis Network, a pan-Canadian, not for-profit oncology network of 16 hospital sites [22, 23]. Informed consent from the participants was not required, as the study was based on retrospective data collection from electronic or paper medical records. Clinicians and hospital employees pre-screened patients based on the inclusion and exclusion criteria listed in the Study Population section. At each site, trained staff, which included personnel responsible for data entry in the PMT program, collected data from patients’ medical records (electronic and paper files). They then documented this data in electronic case report forms specifically designed for the study (OpenClinica), ensuring alignment with the data architecture, variables and dictionary implemented in the PMT registry. Furthermore, they strictly adhered to identical data entry guidelines, which provided clear instructions for precise variable definitions and uniform data handling not only across all study sites but also in alignment with the concurrently developed PMT database. The data was monitored using integrated edit checks, and on-site or remote monitoring was conducted depending on the sites due to the COVID-19 situation. Data lockout and extraction were performed in May 2021, resulting in a deidentified dataset for 70 patients. The available data included demographics, clinicopathology, clinical events, and treatments.

The PMT cancer patient registry, led by Exactis Innovation, was created in 2015 and is currently active across the entire Exactis Network of 16 cancer centers located in 5 provinces in Canada (Quebec, Ontario, New Brunswick, Nova Scotia, and Alberta). This longitudinal oncology registry is an REB-approved initiative in which cancer patients consent to provide access to their medical records for data collection and are prospectively followed throughout their cancer trajectory (NCT02355171). The PMT registry currently aggregates clinical and molecular data from a cohort of over 9,000 Canadian participants and growing. This dataset encompasses a wide array of information including demographics, diagnosis, cancer type, lesion topography and morphology, cancer history, progression and recurrence events, surgery and pathology records (e.g., tumor resection and biopsies), details of systemic chemotherapies and radiotherapies, exhaustive information on biomarkers testing and results, and date of death. The database undergoes updates at a minimum annually, and specific requests for updates can be accommodated to tailor the database for specific studies. The primary cancer cohorts encompassed within the registry include breast, colorectal, lung, prostate, melanoma, and ovarian and, to a lesser extent, endometrial, bladder, and pancreas. PMT coordinators at each participating site enroll patients in the PMT program, collect data from patients’ hospital medical records (electronic and paper files), and enter the deidentified data into the centralized, standardized, and secured PMT database. In 2020, the number of patients in the PMT registry matching the eligibility criteria of the present study was inadequate and the chart review method described above was the preferred method to achieve the study objective. In 2023, medical data from patients enrolled in the PMT registry was included to assess the evolution of standard of care treatment patterns across the Exactis Network.

### Study population

Study participants met the following inclusion criteria: 1) Male or female patients (≥18 years of age); 2) Diagnosed with stage IV breast cancer (*de novo* or progression); 3) HER2+ status in the metastatic setting; 4) Received at least three lines of active anti-cancer drugs due to disease progression in the metastatic setting; 5) Started third-line therapy prior to 31 October 2018 and after 1st November 2018 for the CR cohort and the PMT cohort, respectively. The only exclusion criterion applied was that patients could not have received an investigational anticancer agent in third-line settings or higher. Patients enrolled in clinical trials during the first and second lines of treatment were eligible. Patient selection in both cohorts was exclusively determined by the stringent criteria cited above.

**FIGURE 1:**
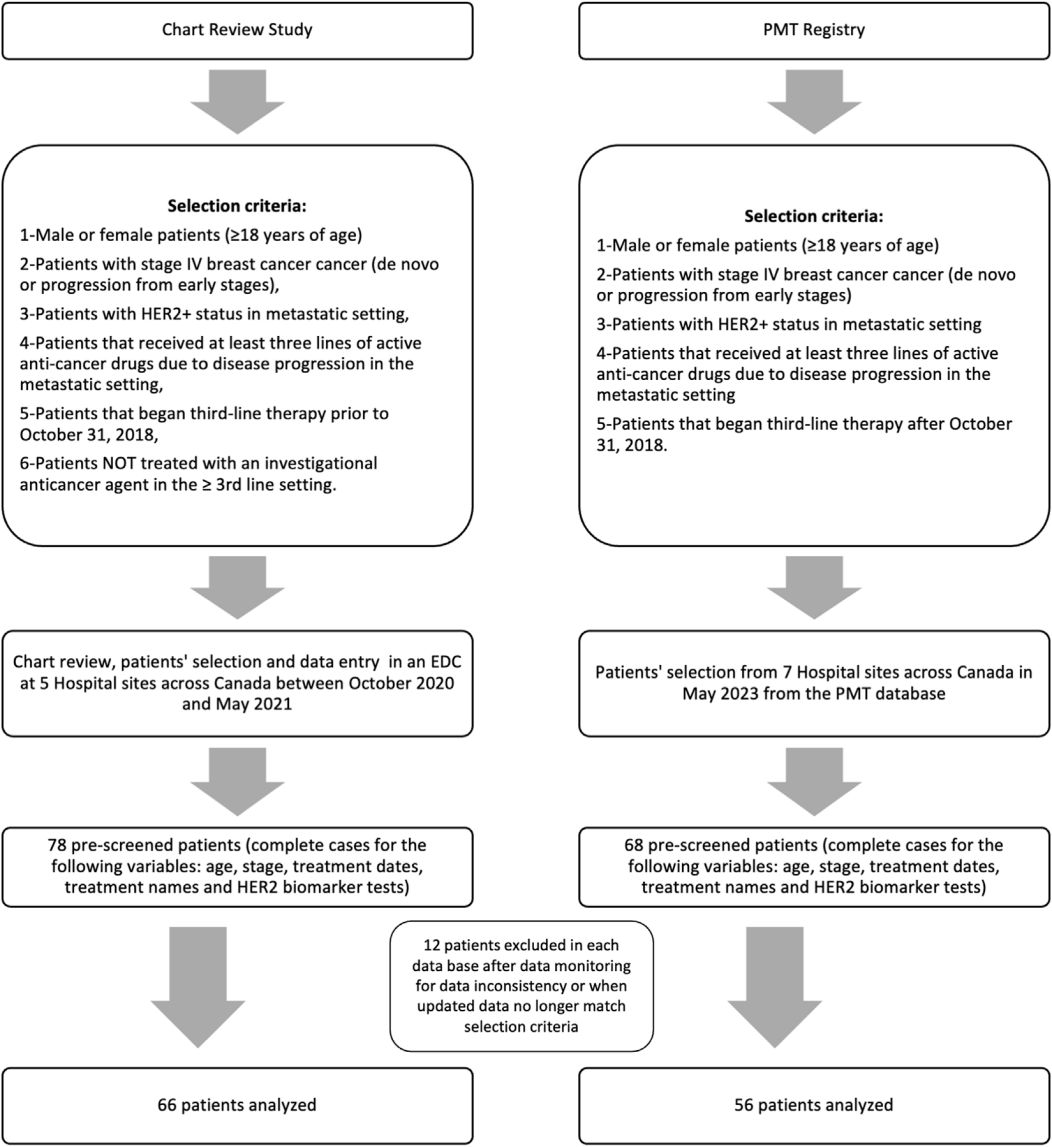
Flow chart of the study design.

The chart review (CR cohort) was performed across 5 different sites within the Exactis Network to identify eligible patients. Seventy-eight patients were pre-screened for the study, and a total of 66 patients with HER2+ mBC met the eligibility criteria following monitoring (Figure 1). Participant medical data were collected and entered in a dedicated electronic data capture system between November 2020 and April 2021 at 5 Exactis Network sites: Jewish General Hospital (JGH), Centre Hospitalier Universitaire de Sherbrooke (CHUS), The Moncton Hospital (TMH), Dr. Georges-L.-Dumont University Hospital Centre (GLD) and The Ottawa Hospital (TOH). Patient distribution by site is available in Supplementary Table S1.

The PMT cohort includes consented patients treated at 8 different sites within the Exactis Network: JGH, CHUS, TMH, TOH, the Centre Hospitalier Université de Québec (CHUQ), the Centre Hospitalier de l’Université de Montréal (CHUM), CIUSSS de la Mauricie-et-du-Centre-du Québec (MCQ) and Sunnybrook Hospital (SBH). Sixty-eight patients met the eligibility criteria of the study, 12 patients with incomplete treatment dates or inconsistent data were removed, and 56 patients were analyzed. Data were extracted in July 2023 from the centralized PMT database.

Given our study’s primary objective, which was to assess the third-line treatment landscape of HER2+ mBC patients in Canada, key factors for patient population selection in both cohorts included stage, treatment start and end dates, treatment names, and HER2 biomarker testing information. It is noteworthy that the patients included in our analysis had complete information for these critical variables. Considering that missing data in these variables in patient’s chart is considered a random event, it is not indicative of specific population selection.

As all patients met the inclusion criteria of having received at least three lines of therapy, they all received treatment as part of their first, second, and third-line regimens and the patient count remained consistent throughout the analysis of the three treatment lines for the CR cohort.

### Variables and definitions

Breast cancer patients were categorized into subgroups based on hormonal receptor (HR) status, including estrogen receptor (ER), progesterone receptor (PR), and HER2 expression status. Patients included in the study needed to be HER2 positive defined by a 3+ HER2 immunohistochemistry (IHC) score or a 2+ HER2 IHC score and a positive HER2 *in situ* hybridization (ISH) result. Patients could be either with HR+ (ER+ and/or PR+) or HR- (ER- and PR-).

A line of therapy (LoT) in the metastatic setting was defined as the use of a single or a set of systemic anti-cancer drugs, used within the initial 28-day period after treatment initiation. In the present study, the first LoT started at the first dose received after the metastatic diagnosis, and a new LoT started if one of the following conditions were met: 1) change in anti-HER2 specific agents due to progression or toxicity, and 2) change in chemotherapy due to progression. The addition or removal of hormonotherapy during a line was not considered as a change of line. Modification of the chemotherapy received in combination with anti-HER2 specific agents was considered a change of line only if associated with reported disease progression.

### Statistical analysis

Continuous variables were described using the number of observations (N) and the median when applicable. Categorical variables were described by the number of observations (N) and the frequency (%) of each category. No statistical tests were performed on the descriptive analysis on treatments received in each cohort. Missing data was encountered in the demographic data and was entered as unknown or missing. The value unknown is represented in tables including descriptive summaries. No imputation was performed.

## Results

### Demographics and clinicopathological characteristics of the cohort

The chart review (CR) cohort consisted of 66 patients with a median age of 63.9 years at the time of metastasis diagnosis (Table 1). Among them, 37 (56.1%) patients received treatment in Quebec, while 15 (22.7%) and 14 (22.2%) were treated in Ontario and New Brunswick provinces, respectively. Sixteen (24.2%) patients were initially diagnosed at stage IV, and 71% of the patients had more than one metastatic site. Brain metastases was observed in 57.6% of the patients. Tumor hormone status was positive (ER or PR positive) in 28 patients (42.4%).

**TABLE 1:** Participant’s demographic and clinical characteristics.

Characteristics	CR^a^ N (%)	PMT^b^ N (%)
Number	66	56
Median age (years)	63.9	55
Provinces		
Quebec	37 (56.1)	44 (78.6)
Ontario	15 (22.7)	10 (17.8)
New Brunswick	14 (21.2)	2 (3.6)
Stage at Diagnosis		
I	4 (6.1)	6 (10.7)
II	22 (33.3)	18 (32.1)
III	14 (21.2)	11 (19.7)
IV	16 (24.2)	21 (37.5)
Unknown	10 (15.1)	0
Number of metastatic sites at metastatic diagnosis		
1	19 (28.8)	29 (51.8)
2	30 (45.5)	13 (23.2)
3	8 (12.1)	9 (16)
>3	9 (13.6)	5 (9)
Patients with brain metastases during the course of stage IV BC		
Yes	38 (57.6)	25 (44.6)
No	26 (40.9)	31 (55.4)
Hormone receptor status		
ER and/or PR positive	28 (42.4)	34 (60.7)
ER and PR negative	38 (57.2)	22 (39.3)

^a^ Includes patients having started third-line treatment prior to 31 October 2018.

^b^ Includes patients having started third-line treatment after 31 October 2018.

The PMT cohort included 56 patients with a median age of 55 years at the time of metastasis diagnosis (Table 1). Among them, 44 (66.7%) patients received treatment in the province of Quebec, while 10 (15.2%) and 2 (3.1%) were treated in the province of Ontario and New Brunswick, respectively. Twenty-one (37.5%) patients were initially diagnosed at stage IV, and 48% of the patients had more than one metastatic site. Brain metastases were observed in 44.6% of the patients. Tumor hormone status was positive (ER or PR positive) in 34 patients (60.7%).

### Real-world pattern of the first two lines of therapy

Although the focus of this study is the analysis of real-world treatment patterns in third-line therapy received by HER2+ metastatic breast cancer (mBC) patients, we analyzed the first two lines of therapy received by the 66 patients of the CR cohort, to confirm that the Canadian practices align with international guidelines in this setting. Our results showed that the standard of care for the first-line treatment involved trastuzumab as an anti-HER2 targeted therapy in combination with chemotherapy drugs, with 60.6% of the patients falling into this category. Only 2 (3%) patients received targeted therapy without chemotherapy, and a single patient (1.5%) received chemotherapy without targeted therapy (Table 2). Among the patients receiving trastuzumab, the majority (*n* = 42, 63.6%) received this treatment prior to Health Canada approval of pertuzumab (May 2013). Conversely, among the patients who started first-line treatment after this date (*n* = 31), the majority (*n* = 22, 71%) received the combination of trastuzumab and pertuzumab, while 7 (23%) received trastuzumab combined with chemotherapy, indicating a change in practice in Canada following the availability of pertuzumab (Supplementary Figure S1). Lapatinib was the only other anti-HER2 specific agents given as a first-line treatment, occurring in 2 patients (3%). Taxanes (abraxane, docetaxel, paclitaxel) were the most commonly used chemotherapy agents in 60 (90.9%) patients. Hormone therapies were also used in combination with targeted therapies and chemotherapy in 8 patients (12.1%) (data not shown).

**TABLE 2:** Treatment combination distribution in first-line (CR cohort, defined as having started third-line treatment prior to 31 October 2018).

	Trastuzumab	Trastuzumab + Pertuzumab	Lapatinib	None^a^	Total
Abraxane, docetaxel, paclitaxel and vinorelbine	38 (57.6)	21 (31.8)	1 (1.5)	0	60 (90.9)
Anthracyclines (Doxorubicine)	1 (1.5)	0	0	0	1 (1.5)
Antimetabolite (Capecitabine)	1 (1.5)	0	1 (1.5)	0	2 (3)
Alkaloids+Alkylating agent (AC)	0	0	0 (0)	1 (1.5)	1 (1.5)
None^a^	2 (3)	0	0	0	2 (3)
Total	42 (63.6)	21 (31.8)	2 (3)	1 (1.5)	66 (100)

^a^ None refers to patients who did not receive anti-HER2 specific agents or chemotherapy but may have received other targeted or endocrine therapies.

For each category, data is presented as the count and the corresponding percentage (%) of the total.

As a second-line therapy, trastuzumab and the combination of trastuzumab and pertuzumab were given to 29 (43.9%) and 3 (4.5%) patients, respectively (Table 3). Lapatinib represented 4.5% of the targeted therapies in this setting, while T-DM1 was chosen in 19 (28.8%) cases. In unique cases, T-DM1 was given in combination with tucatinib (*n* = 1, 1.5%) or trastuzumab (*n* = 1, 1.5%). Among the subgroup of patients who started their second-line treatment in 2014 or after (*n* = 32), when T-DM1 was reimbursed in Canada, 21 patients (65.6%) received T-DM1 alone or in combination with tucatinib or trastuzumab, confirming the change in practice in this setting (Supplementary Figure S1). Interestingly, 10% of cases did not receive any targeted therapies in this setting. Similar to the first-line treatment, trastuzumab was mostly combined with taxanes in the second-line treatment. Among the 4 patients who received lapatinib, 3 were given gemcitabine or capecitabine as chemotherapy. T-DM1 was not given in combination with chemotherapy, except in a single case (T-DM1, trastuzumab, and chemotherapy). Hormone therapies were also used in combination with targeted therapies and/or chemotherapy in 4 patients (6.1%) (data not shown).

**TABLE 3:** Treatment combination distribution in second-line (CR Cohort, defined as having started third-line treatment prior to 31 October 2018).

	Trastuzumab	Trastuzumab + Pertuzumab	Lapatinib	T-DM1	T-DM1 + Tucatinib	T-DM1 + Trastuzumab	None^a^	Total
Aabraxane, docetaxel, paclitaxel and vinorelbine	16 (24.2)	2 (3)	0	0	0	1 (1.5)	2 (3)	21 (31.8)
Anthracyclines (Doxorubicine)	2 (3)	0	0	0	0	0	0	2 (3)
Antimetabolite (Gemcitabine, Capecitabine)	7 (10.6)	0	3 (4.5)	0	0	0	7 (10.6)	17 (25.7)
Alkylating agent (Carboplatin)	1 (1.5)	0	0	0	0	0	0	1 (1.5)
Other (Palbociclib)	0	0	0	0	0	0	1 (1.5)	1 (1.5)
None^a^	3 (4.5)	1 (1.5)	0	19 (28.8)	1 (1.5)	0	0	24 (36.4)
Total	29 (43.9)	3 (4.5)	4 (4.5)	19 (28.8)	1 (1.5)	1 (1.5)	10 (15.2)	66 (100)

^a^ None refers to patients who did not receive anti-HER2 specific agents or chemotherapy, but may have received other targeted or endocrine therapies.

For each category, data is presented as the count and the corresponding percentage (%) of the total.

### Real-world patterns of the third-line therapy

In the CR cohort, as the third-line treatment, trastuzumab represented more than half of the targeted therapy regimens given in this setting but was not combined with pertuzumab in any of the patients (Table 4). In 34 patients (51.5%), trastuzumab was given in combination with chemotherapy, mainly taxanes (15, 22.7%), and with gemcitabine or capecitabine (13, 19.7%). Thirteen patients (19.7%) received lapatinib, and 1 (1.5%) patient received lapatinib in combination with trastuzumab and capecitabine. T-DM1 alone was given to 6 patients, representing 9.1% of the entire cohort or 15.4% of patients who started their treatment after 2014 when the drug became available and reimbursed. Nine (13.6%) patients did not receive any anti-HER2 specific agents. None of the patients received hormone therapies in this setting (data not shown).

**TABLE 4:** Treatment combination distribution in third-line (CR Cohort, defined as having started third-line treatment prior to 31 October 2018).

	Trastuzumab	Lapatinib	Lapatinib + Trastuzumab	T-DM1	None^a^	Total
Paclitaxel and vinorelbine	15 (22.7)	0	0	0	4 (6.1)	19 (28.8)
Doxorubicine	2 (3)	0	0	0	0	2 (3)
Gemcitabine, Capecitabine	13 (19.7)	13 (19.7)	1 (1.5)	0	3 (4.5)	30 (45.5)
Alkylating agent (Carboplatin and Cyclophosphamide)	2 (3)	0	0	0	0	2 (3)
Carboplatin + gemcitabine	1 (1.5)	0	0	0	0	1 (1.5)
Alkylating agent + antimetabolite (Gemcarbo)	0	0	0	0	1 (1.5)	1 (1.5)
Non-taxane microtubule inhibitor (Eribulin)	1 (1.5)	0	0	0	1 (1.5)	2 (3)
None^a^	3 (4.5)	0	0	6 (9.1)	0	9 (13.6)
Total	37 (56.1)	13 (19.7)	1 (1.5)	6 (9.1)	9 (13.6)	66 (100)

^a^ None refers to patients who did not receive anti-HER2 specific agents or chemotherapy, but may have received other targeted or endocrine therapies.

For each category, data is presented as the count and the corresponding percentage (%) of the total.

To assess whether the real-world third-line treatment pattern for HER2+ mBC patients had evolved since 2018, we used the PMT registry to collect and analyze RWD from an independent cohort of 56 Canadian patients (Table 1) who started third-line treatment after 31 October 2018. Among the 50 remaining patients (excluding those enrolled in clinical trials), only 12 (24%) received trastuzumab alone (7, 14%) or in combination with chemotherapy (5, 10%), primarily paclitaxel or vinorelbine (3, 6%) (Table 5). This cohort revealed that trastuzumab was also combined with other anti-HER2 therapies in an additional 10 patients (20%), including pertuzumab (5 patients, 10%) patients, pertuzumab and T-DM1 (1 patient, 2%), and tucatinib in combination with capecitabine (4 patients, 8%) cases, including 3 patients with brain metastases (Table 5). These combinations were not observed in the cohort of patients who started their treatment prior to October 2018. In total (PMT cohort) trastuzumab, alone or in combination with other targeted therapies or chemotherapy, was given to 22 (44%) patients. Importantly, T-DM1 alone was chosen in 18 (36%) cases, and 1 (2%) additional patient received T-DM1 in combination with pertuzumab, highlighting a significant increase in the proportion of patients treated with T-DM1 compared to the previous assessment.

**TABLE 5:** Treatment combination distribution in third-line (PMT Cohort, defined as having started third-line treatment after 31 October 2018).

	Trastuzumab	Trastuzumab + pertuzumab	Trastuzumab + pertuzumab + T-DM1	Trastuzumab + Tucatinib	T-DM1	T-DM1 + Pertuzumab	T-DXd	Lapatinib	None^a^	Total
Paclitaxel and vinorelbine	3 (6)	2 (4)	0	0	0	0	0	0	1 (2)	6 (12)
Gemcitabine, Capecitabine	1 (2)	0	0	4 (8)	0	0	0	2	2 (4)	9 (18)
Alkylating agent + antimetabolite (Gemcarbo)	1 (2)	0	0	0	0	0	0	0	0	1 (2)
None^a^	7 (14)	3 (6)	1 (2)	0	18 (36)	1 (2)	1 (2)	0	3 (6)	34 (68)
Total	12 (24)	5 (10)	1 (2)	4 (8)	18 (36)	1 (2)	1 (2)	2 (4)	6 (12)	50 (100)

^a^ None refers to patients who did not receive anti-HER2 specific agents or chemotherapy, but may have received other targeted or endocrine therapies.

For each category, data is presented as the count and the corresponding percentage (%) of the total.

On the contrary, we observed a sharp decrease in the number of patients receiving Lapatinib (2, 4%). The use of T-DXd as a third-line treatment was attempted in a single patient (2%). Similarly, to the CR cohort, 6 (12%) patients did not receive any anti-HER2 specific agents. Hormone therapies were used in combination with chemotherapy in 2 cases (4%).

**FIGURE 2:**
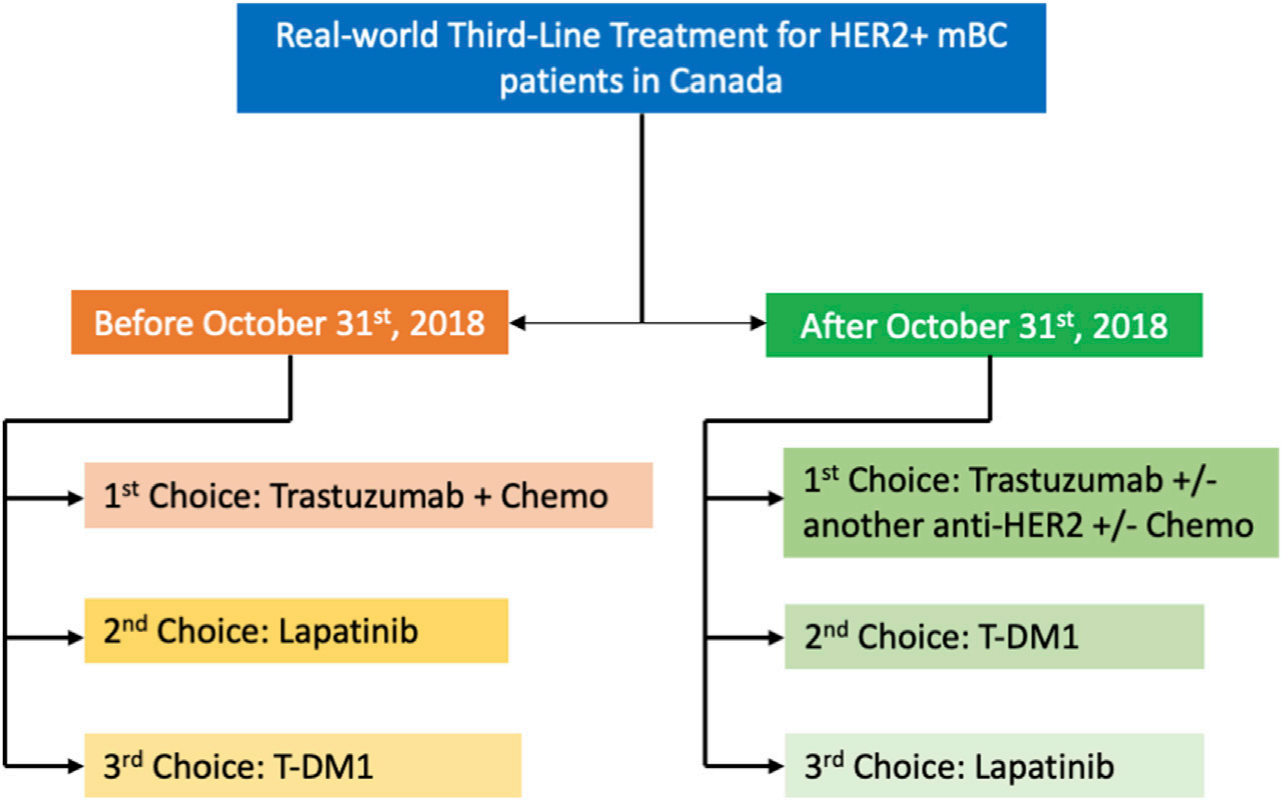
Real-world third-line treatment pattern for HER2+ mBC patients in Canada. Flowchart summarizing third line treatment options in Canada inferred from the analysis of the CR cohort in orange (third-line treatment started prior to 31October 2018) and the PMT cohort in green (third-line treatment started after 31 October 2018 but prior to public reimbursement across all provinces of tucatinib, neratinib and T-DXd).

The analysis of real-world third-line treatment given after October 2018 revealed the emergence of new therapeutic strategies in Canada, involving combinations of different anti-HER2 agents, a significantly higher representation of T-DM1, and a reduction of lapatinib use (Figure 2). Overall, the most frequently chosen drug in this setting remained trastuzumab, either alone or in combination with another anti-HER2 agent or chemotherapy.

## Discussion

In Canada, RWD pertaining to oncology is primarily collected by individual provinces and territories for administrative purposes. Increasingly, these datasets are being leveraged to generate real-world evidence to inform healthcare policy decisions. The 97 provincial databases established in Canada are held by various data custodians [24]. Thus, the accessibility, governance, and availability of these datasets vary significantly among provinces, thereby impeding the aggregation of patient-level data for nationwide assessments, a capability observed in other countries. In addition to these data access challenges, Canadian administrative databases suffer from a noteworthy limitation: the inconsistency in the types of variables collected across provinces. This inconsistency leads to difficulties in linking and interoperability of data, as well as issues with data coverage and availability over time. Moreover, these databases often exhibit gaps in information related to oral oncology treatments and biomarker testing results. The PMT registry stands as a pioneering initiative in Canada, offering a comprehensive, longitudinal, and standardized repository of real-world oncology data, on multiple cancer types, sourced from 16 distinct sites spanning 5 provinces. This platform’s significance is underscored by Exactis’ recent inclusion as a Network Collaborator within the Canadian Agency for Drugs & Technologies in Health (CADTH) Post-market Drug Evaluation (PMDE) Program to help provide evidence-based responses to queries and concerns raised by decision-makers at the federal, provincial, and territorial levels concerning drugs approved for use in Canada.

In this study we harnessed the PMT registry to broaden our assessment of the post-second-line treatment landscape for HER2+ mBC patients which relied on a chart review approach. This expansion of our research scope builds on our initial investigation in 2020, a time when the PMT registry had not reached a sufficient level of maturity, which permitted us to infer evolutionary trends in patient management.

By retrospectively collecting the initial data from medical records of 66 patients who started third-line therapy prior to October 2018 (CR cohort), we first observed a significant evolution in treatment strategies in Canada following availability of pertuzumab and T-DM1. These developments align with international guidelines and indicate that healthcare practitioners in Canada tend to embrace new treatments promptly, particularly in the context of metastatic conditions, as soon as they become available.

We also observed an overall increased heterogeneity of therapies used in clinical practice starting at second-line treatment and beyond. More specifically, following the internationally well-established first-line treatment regimen of trastuzumab plus taxanes ± pertuzumab (95%), 65.6% of patients treated from 2014 received T-DM1 in second-line, which was, until publication of the results of the DESTINY Breast-03 trial in 2021, the recognized standard of care regimen. Not surprisingly, the type of treatment received during third-line was more heterogeneous, with 56% receiving trastuzumab, 20% receiving lapatinib, 14% receiving chemotherapy alone, and 10% receiving T-DM1 (or 16.6% if only patients treated during or after 2014 were considered), highlighting a lack of consensus in how to treat these patients after a second progression and reflecting the fact that data were collected from several provinces with different access to lapatinib during the course of the disease. While we acknowledge the patient-specific nature of administrated line of treatment, it is important to recognize that customization based on patient treatment response was not investigated in our study. Unfortunately, treatment response data were not captured in the databases.

Three years later, we performed a new assessment of the third-line landscape in the same indication, using data from Canadian patients who received third-line therapy after October 2018 (PMT cohort). The results revealed novel targeted therapy combinations such as trastuzumab with pertuzumab and trastuzumab with tucatinib and capecitabine in this indication. Health Canada approved Tucatinib in 2020 in combination with capecitabine and trastuzumab in patients who have received prior trastuzumab, pertuzumab, and T-DM1, based on the HER2CLIMB trial results that showed for the first time that systemic targeted therapy could control brain metastases and improve patient overall survival. The small proportion of patients receiving this regimen in our PMT cohort is expected as tucatinib became publicly reimbursed only in November 2022 in Quebec and in February 2023 in Ontario. On the other hand, the use of T-DM1 became more than twice as frequent as previously observed, highlighting the strategy shift from lapatinib to T-DM1. This is in agreement with the EMILIA trial [16] results as well as with an RWE study using data from Flatiron Health database that showed a longer OS for patients treated with T-DM1 versus Lapatinib as second-line therapy [24, 25]. Nonetheless, trastuzumab alone or in combination with other targeted therapies or chemotherapies remains the preferred choice as third-line in Canada. Not surprisingly, access to T-DXd was limited, since the price negotiation of T-DXd by the Pan-Canadian Pharmaceutical Alliance (pCPA) occurred in May 2023.

We acknowledge that drug efficacy and effectiveness play a crucial role in clinical decision-making. Although our study did not extensively explore these aspects, we understand that healthcare professionals base their decisions on a blend of factors, including drug availability, clinical trial data, patient characteristics, and individual patient responses. A larger patient cohorts would certainly be valuable to explore the multifaceted factors influencing treatment selection in greater depth, including the roles of efficacy and effectiveness.

The third-line landscape described in this study will likely evolve in the next few years with the upcoming public reimbursement across Canadian provinces of Neratinib, Tucatinib, and T-DXd. The expected replacement of T-DM1 by T-DXd as the standard second-line therapy for HER2+ mBC will subsequently impact treatment sequence and the choice of a third-line after progression. Based on the results of the phase III SOPHIA trial [26], Margetuximab, a second-generation anti-HER2 monoclonal antibody, was recently approved in the USA for use in combination with chemotherapy as treatment of previously treated HER2+ mBC and may soon be a novel therapeutic option for Canadian patients. In this context, RWD collection will be instrumental to assess the change of Canadian practices, evaluate real-world efficacy of each drug, and optimize treatment sequence, understand the patterns of cross-resistance among different available anti-HER2 agents, and possibly support a specific approach as third-line therapy. *De novo* and acquired mechanisms of resistance are some of the most frequent challenges encountered in the era of targeted therapies, and increasing the diversity of anti-HER2 therapies available will alleviate this problem and should result in improving patients’ durable response and survival.

There are several limitations in the current observational study that need to be acknowledged. These include: 1) the small number of patients included in the study prevented the investigation of real-world efficacy and associated patient outcomes of the different third-line treatments; Confirmation of our results using a larger cohort is necessary to provide more robust insights into the Canadian treatment 2) the representation of only 3 provinces of Canada, with a substantial majority of patients in both cohort originating from the province of Quebec, does pose limitations on the generalization of the observation at the scale of the country; The Exactis Network is actively pursuing the expansion of its cancer registry, both within the provinces already participating and in additional provinces. The Network has initiated the enrollment of HER2 positive BC patients in Alberta and Nova Scotia. Ongoing efforts are underway to include patients from Manitoba, Saskatchewan, Prince Edouard Island and Newfoundland. 3) The utilization of two distinct databases to compare cancer treatments administered over two distinct time periods may introduce potential biases related to variations in patient populations and differences in data quality. Notably, differences in the median age of patient populations were observed between the two cohorts, with the CR cohort displaying a median age of 64, compared to 55 in the PMT cohort. This age disparity can be attributed to the well-documented phenomenon of patient consent bias in clinical research, where younger individuals are more likely to participate. However, it’s important to underscore that the primary objective of this study was to provide a descriptive analysis of the evolving treatment landscape rather than an assessment of patient outcomes, for which a 9 years age differences could indeed have more substantial implications. Regarding data quality, it is important to highlight the high level of standardization of data capture in both cohorts. This standardization was achieved through uniform data collection practices, including the design of the EDC system for the CR cohort, which closely aligned with the data architecture, variables, and dictionary employed in the PMT registry. Additionally, the involvement of the same operational team for data entry ensured consistency, with strict adherence to identical data entry guidelines. Additionally, a centralized monitoring process was performed before each database’s closure to minimize inconsistencies and missing data. This data capture standardization across sites and provinces is a unique feature in Canada, ensuring comparable data quality levels, mitigating the likelihood of introducing substantial bias in this study.

Overall, our real-world study confirmed that there is still no consensus in Canada for the post second-line treatment of HER2+ mBC patients, and its evolution is directly linked to new drug access. The delay for Canadian patients to access new publicly reimbursed medicine such as neratinib, tucatinib, and T-DXd is concerning and highlights the urgent need for the modernization of the system to create additional opportunities for reimbursement and faster drug access to all Canadians.

## Data Availability

The datasets presented in this article are not readily available because clinical patient-level data are not publicly available. Requests to access the datasets should be directed to mmarques@exactis.ca.

## Supplementary material

SUPPLEMENTARY FIGURE S1 Real-world first two lines of treatment pattern for HER2+ mBC patients in Canada.

SUPPLEMENTARY TABLE S1 Distribution of patients by sites.

## Abbreviations

BC, Breast cancer; BM, Brain metastases; CHUS, Centre Hospitalier Universitaire de Sherbrooke; CI, Confidence Interval; DCR, Disease control rate; ECOG, Eastern Cooperative Oncology Group; ER, Estrogen receptor; FISH, Fluorescence In Situ Hybridization; GLD, Georges-L.-Dumont University Hospital Centre; HER2, Human epidermal growth factor receptor 2; HER2+, Human epidermal growth factor receptor 2 positive; HR, Hazard ratio; HRU, Health resource utilization; IHC, Immunohistochemistry; L, Line; LoT: line of therapy; JGH, Jewish General Hospital; mBC, metastatic Breast Cancer; N, Number; PR, Progesterone receptor; RWD, Real world data; T-DM1, Trastuzumab-DM1 or ado-trastuzumab emtansine; TMH, The Moncton Hospital; TNT, Time-to-next treatment; TOH, The Ottawa Hospital.
