# Supplementary material for: Third-line treatment patterns in HER2-positive metastatic breast cancer: a retrospective analysis of real-world data in Canada
Source: J Pharm Pharm Sci. 2023 Dec 13;26:12078. doi: 10.3389/jpps.2023.12078 (PMC10751338; doi:10.3389/jpps.2023.12078)
Supplement: Supplementary file 1 [file DataSheet1.PDF]

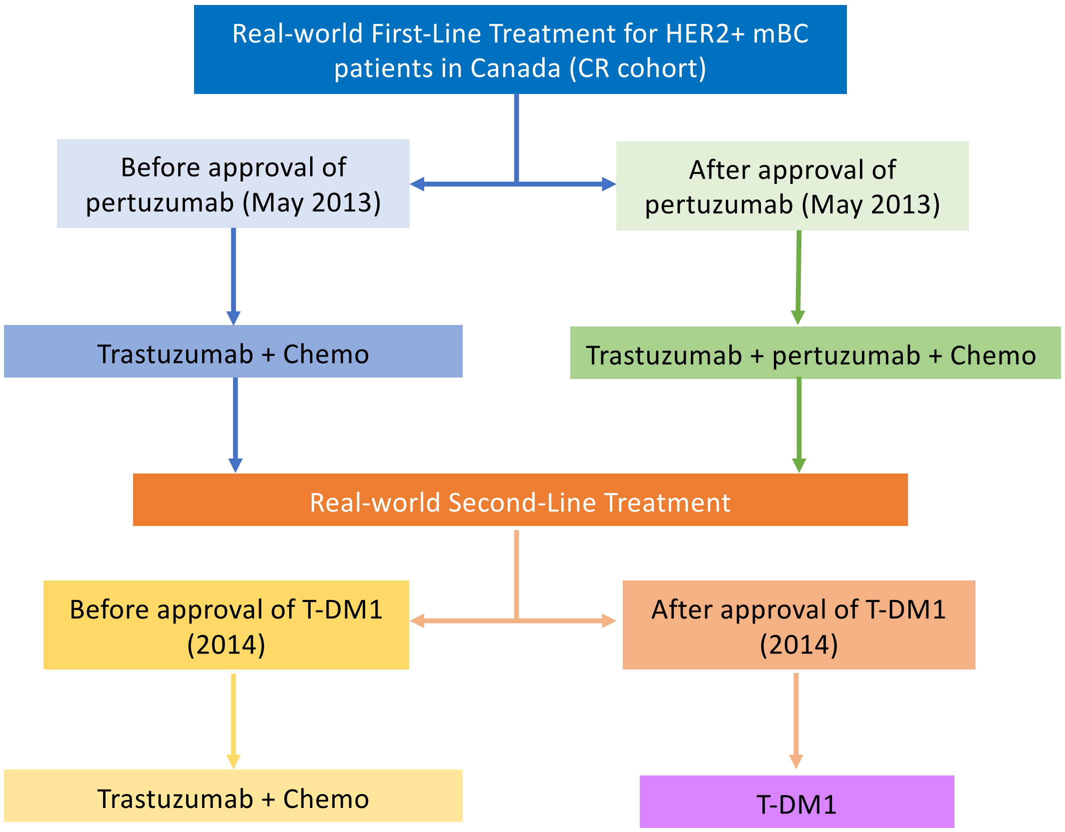

Figure S1: Flowchart summarizing the first two lines of treatment sequence in Canada inferred from the analysis of the CR cohort.
